# Using Ion-Selective Electrodes to Study the Drug Release from Porous Cellulose Matrices

**DOI:** 10.3390/pharmaceutics4030366

**Published:** 2012-08-07

**Authors:** Hossein Vakili, Natalja Genina, Henrik Ehlers, Johan Bobacka, Niklas Sandler

**Affiliations:** 1 Pharmaceutical Sciences Laboratory, Department of Biosciences, Abo Akademi University Artillerigatan 6 A, 20520, Turku, Finland; Email: natalja.genina@abo.fi (N.G.); hehlers@abo.fi (H.E.); niklas.sandler@abo.fi (N.S.); 2 Analytical Chemistry Laboratory, Department of Chemical Engineering, Biskopsgatan 8, 20500, Turku, Finland; Email: jbobacka@abo.fi

**Keywords:** ion-selective electrodes, potentiometry, UV spectrophotometry, propranolol hydrochloride, lidocaine hydrochloride, polymer film, porous substrates

## Abstract

Polyvinyl chloride (PVC)-based solid-contact ion-selective electrodes (SC-ISEs), responding to propranolol hydrochloride (Pr^+^) and lidocaine hydrochloride (Ld^+^) cations as the model drugs with potassium tetrakis(4-chlorophenyl) borate (KTpClPB) as the ion exchanger, were studied. Different drug-polymer solutions were prepared with the model drugs, using different blend ratios of ethylcellulose (EC) and hydroxypropyl cellulose (HPC). Two different solid dosage forms were used. Polymer films were produced by solvent casting method and drug containing porous cellulose samples were prepared by depositing the drug-polymer solutions onto filter paper substrates. The quality of the electrodes and the release profile of Pr^+^ and Ld^+^ were investigated with the potentiometric method. The results were compared to UV spectrophotometry. The electrodes were found to be sensitive, precise and functional with a Nernstian behavior over the range of 1.0 × 10^−3^–3.1 × 10^−6^ M (9.2 × 10^−4^–3.0 × 10^−1^ mg/mL) and 1 × 10^−3^–2 × 10^−6^ M (5.4 × 10^−4^–2.7 × 10^−1^ mg/mL) at 25 °C for Pr^+^ and Ld^+^ sensitive electrodes, respectively. The dynamic response time for the electrodes was less than 10 s. The Pr^+^ release from porous filter paper was always higher than its equivalent film formulation. Also, lidocaine had higher and faster release from the samples with higher drug concentration. The comparison of the two analytical methods showed near identical results. The ISEs provided a powerful and flexible alternative to UV method in determination of drug release from porous cellulose substrates in a small scale dissolution testing.

## 1. Introduction

Ion-Selective Electrodes (ISEs) have been used for several decades to determine inorganic ions [1,2]. Ever since Baum *et al*. [3,4,5] introduced the organic sensitive sensors in the seventies, apart from many studies about their significant role in analytical, environmental, industrial and biological fields, also a great amount of research has been done about the application of ISEs in the field of pharmaceutical sciences [6,7,8,9]. For example Coşofreţ *et al*. [10] studied a polyvinyl chloride (PVC) membrane selective towards various drugs and excipients and also in particular for determination of phenytoin in pharmaceutical formulations [11]. 

The conventional methods in pharmaceutical applications for determination of the drug concentration such as gas chromatography, high performance liquid chromatography (HPLC), UV spectrophotometry and other expensive and sophisticated methods have several limitations. In particular, as for UV spectrophotometry, the turbidity caused by the formation of suspensions and colloids during dissolution, presence of UV active excipients, narrow detection range, air bubbles in the beam path, and relative poor sensitivity for certain drugs can be problematic [12,13]. More recently, in-line (*in situ*) UV fiber optic technology, where no pumps and sampling are required, has been introduced. The new automation of dissolution systems leads to much faster and more continuous profiles, however, these methods cannot be used for every product, because e.g. large amounts of undissolved particles may cause scattering of the UV light. Moreover, as opposed to off-line and on-line measurements, filtration is not possible. Therefore, it is essential to introduce new technology platforms to develop new approaches and reliable tools for dissolution analysis of a variety of the drug products in different types of drug-delivery systems [14]. In this context, potentiometric methods are promising and may offer many beneficial functional properties.

Pharmaceutical applications of ISEs include determination of the content uniformity and dissolution profiles of different active pharmaceutical ingredients (APIs). ISEs are able to measure the selective activity of a variety of APIs directly and continuously, e.g., each 10 s, resulting in much more informative dissolution profiles. The measurements can be done in most cases without pretreatment steps or prior separation of the APIs from the formulation matrix. The advantages of ISEs are e.g., simple and inexpensive design, adequate accuracy and extensive linear range resulting in accurate data at the early stages of dissolution. In addition, low detection limit, easy maintenance, insensitivity towards many excipients and suspensions, long durability and fast response can be mentioned. The fast response gives the ISEs a great advantage in measuring the dissolution profiles of immediate release dosage forms [12]. This important information is useful in optimization of formulations for pharmaceutical dosage forms [15]. On the other hand, ISEs may suffer from reproducibility and the long-term stability (drifting potential) problems, resulting from poorly defined contact or mechanism of charge transfer between the membrane layer and the measuring device [16]. To improve the detection limit, stability and reproducibility of ISEs, the liquid internal half-cell in conventional ISEs can be replaced by a low diffusivity membrane matrix with low water uptake [17]. The selectivity of the electrodes toward various lipophilic drug compounds can be increased by adding lipophilic ionic additives to the highly plasticized membrane structure [18]. Tetraphenylborate additives have been used as active ion pairing agents to produce negative charge at the surface of the electrodes for the purpose of complexation with local anesthetics cations [19]. Carbon cloth was recently used as ion-to-electron transducer with mechanical flexibility and high specific surface area, causing a larger double-layer capacitance and lower impedance at the interface between the electrode and the membrane. This results in a simple design and manufacturing of a very robust ISE [20].

The main aim of this study was to prepare PVC matrix-based SC-ISEs for monitoring lidocaine hydrochloride and propranolol hydrochloride release from porous cellulose matrices. The selectivity and quality of the electrodes were investigated, and the potentiometric results were compared to UV spectrophotometric analysis. 

## 2. Experimental Section

### 2.1. Materials

Propranolol hydrochloride, lidocaine hydrochloride monohydrate and ethylcellulose (EC) were purchased from Sigma (St. Louis, MO, USA), hydroxypropyl cellulose (HPC) was purchased from Shin-Etsu (Shin-Etsu, Japan). Ethanol (≥96.1%, Etax A, Altia OYj, Finland) was used as the solvent for drug-polymer solutions. Polyvinyl chloride (PVC), 2-nitrophenyl octyl ether (NPOE), potassium tetrakis(4-chlorophenyl) borate (KTpClPB) and tetrahydrofuran (THF) were Selectophore reagents from Fluka (Hauppauge, NY). Carbon cloth (Kynol^®^ activated carbon fabric ACC-5092-20) was purchased from Kynol Europa GmbH (Hamburg, Germany). Uncoated filter paper (138 g/m^2^) was purchased from Whatman International Ltd (Maidstone, England).

### 2.2. Working Principle of Electrodes

To develop ISEs with a potential difference dependent only on a single activity of target cation C^+^, two ions of oil soluble X^−^ and mainly water soluble N^+^ also should be present [18]. The lipophilic ion pair salt of N^+^X^−^ is incorporated in the sensor membrane. Then the electrodes are conditioned with the solution containing the target cation C^+^. Since C^+^ is usually much more lipophilic than N^+^, it replaces N^+^ in the membrane to FORM C^+^X^−^ while N^+^ is released to the test solution. Once the activity of C^+^ in the membrane becomes stable, the electrodes are capable to detect the activity of C^+^ in the test solution [21]. The lifetime of the electrodes is mainly related to the leaching of the membrane components into the surrounding solutions.

### 2.3. Preparation of ISEs

**Figure 1:**
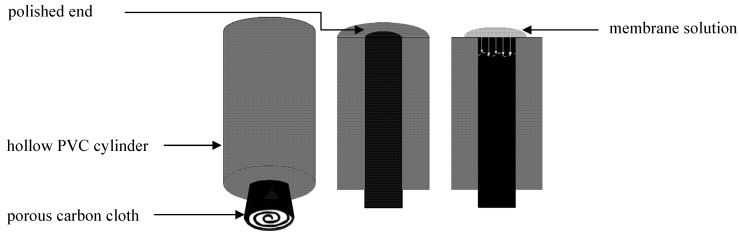
Schematic picture of carbon cloth-based electrodes. **Left**: Hollow PVC cylinder with carbon cloth in the center of the electrode; **Middle**: Cylinder shown from top with the polished end; **Right**: Cylinder shown with membrane solution that has been placed on the top of the polished end.

The composition of the membrane was as following: 33% PVC, 66% *O*-NPOE and 1% KT*p*ClPB (*w*/*w*). The membrane solution consisted of 20% (*w*/*w*) of the “dry” part (PVC, *O*-NPOE, KTpClPB) and 80% of THF as the solvent. Rectangular pieces (7 × 2 cm^2^) of carbon cloth as the electrically conductive substrate were cut and rolled up and mounted inside a PVC cylinder with a central cylindrical hole of 5 mm diameter. The total amount of 450 µL of the resulting clear mixture (membrane cocktail/solution) was drop-casted on the surface of each carbon cloth electrode in portions of 100 µL in every 30 min (Figure 1). The obtained membrane was allowed to air-dry at room temperature for 2 days. Three electrodes were conditioned in 1.0×10^−3^ M propranolol hydrochloride + 10^−2^ M HCl (pH 2.0) and another three electrodes were conditioned in 1.0×10^−3^ M lidocaine hydrochloride + 10^−2^ M HCl (pH 2.0). 

### 2.4. Preparation of the Drug-Containing Solid Dosage Forms

Nine different types of drug-polymer solutions consisting of propranolol (20%) or lidocaine (20% or 40%) and polymers were prepared. The polymers consisted of EC and HPC with EC:HPC mass ratios of (1:0, 1:1 and 0:1). The mixed powder of the drug and polymers (1 g) were dissolved in 5 mL of ethanol at 60 °C by using ultrasound bath for 15 min. Polymer films were made by casting 1 mL of the above mentioned drug-polymer solutions on a Teflon mold (inner diameter of 20 mm, 10 mm deep). For the second formulation, 0.5 mL of the drug-polymer solutions was manually deposited with a syringe onto the circular application area of the 28 mm diameter filter paper. Three parallel samples were prepared in each case. The samples were left to dry in a desiccator containing silica gel at room temperature for 24 h before starting the measurement. 

### 2.5. Potentiometric and UV Spectrophotometric Measurements

All the potentiometric measurements were carried out in a 100 mL plastic flask while stirring at 300 rpm with a IKA^®^ RCT Hotplate magnetic Stirrer (Staufen im Breisgau, Germany) at room temperature. Three parallel ISEs for each drug were dipped into the test solution in the presence of a conventional single junction reference electrode: Ag/AgCl/ 3 M KCl (Metrohm, Switzerland). The measurements were performed by using Lawson labs, Inc. EMF 16 Interface (Phoenixville Pike, USA) and an electrochemical cell of this type:

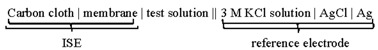


All the off-line UV spectrophotometric measurements were carried out in the same conditions as potentiometric measurements. The measurements were performed using a UV-VIS spectrophotometer (*λ* = 289 nm; PerkinElmer, Lambda 25, USA). One substrate at the time was used for each analysis. At pre-determined time intervals, 1.5 mL samples were manually withdrawn and were added back into the dissolution flask after the measurements. 

## 3. Results and Discussion

### 3.1. The Quality of ISEs

#### 3.1.1. Calibration Curves

**Figure 2:**
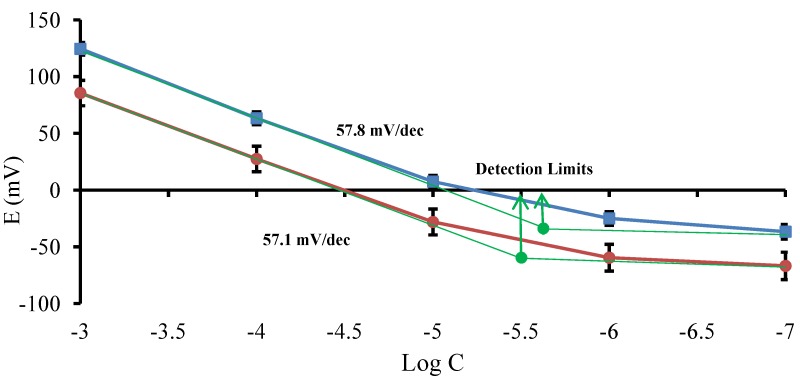
Comparison of the calibration plots after 4 weeks of conditioning in propranolol hydrochloride (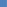) and lidocaine hydrochloride (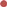). Mean ± SD values are shown (*n* = 3).

The average potential responses of the selective electrodes at different concentrations of model drug cations indicated a Nernstian behavior from 1.0 × 10^−3^–1.0 × 10^−5^ M for both drugs (Figure 2). The slopes of the calibration curves were 57.8 and 57.1 mV decade^−1^ for propranolol and lidocaine electrodes, respectively. The lower detection limits of the electrodes were determined by intersection of the two tangents of each end of the calibration curves. The detection limits were 3.1 × 10^−6^ M and 2 × 10^−6^ M for propranolol and lidocaine electrodes, respectively. The potential stability of the electrodes in the solutions of 1.0 × 10^−3^ M and 1.0 × 10^−4^ M of each drug in the constant background of 1.0 × 10^−2^ M HCl were also investigated for 6 h and the results showed less than 3.0 mV drift in potential response of the electrodes, which is quite satisfactory (between 0%–5% in 6 h). The electrodes were calibrated and their repeatable functionality was proved for more than 6 months in the course of this study by taking them out of the conditioning solution and putting them back in after the measurements.

#### 3.1.2. Response Time

**Figure 3:**
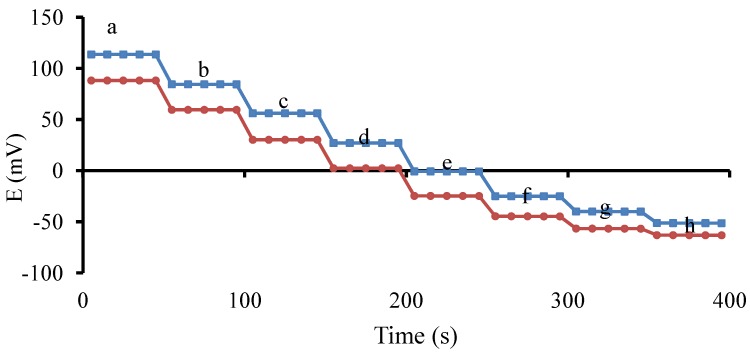
Dynamic response times of the electrodes for step change in concentration of propranolol hydrochloride (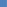) and lidocaine hydrochloride (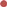). (**a**) 1.0×10^−3^ M; (**b**) 1.0×10^−3.5^ M; (**c**) 1.0×10^−4^ M; (**d**) 1.0×10^−4.5^ M; (**e**) 1.0×10^−5^ M; (**f**) 1.0×10^−5.5^ M; (**g**) 1.0×10^−6^ M and (**h**) 1.0×10^−7^ M.

The dynamic response times of the electrodes were recorded by changing the solution concentration from 1.0 × 10^−3^ M to 1.0 × 10^−7^ M (Figure 3). The response was fast (<10 s) within the entire investigation range, indicating that interface process reached its equilibrium rapidly.

#### 3.1.3. Effect of pH

**Figure 4:**
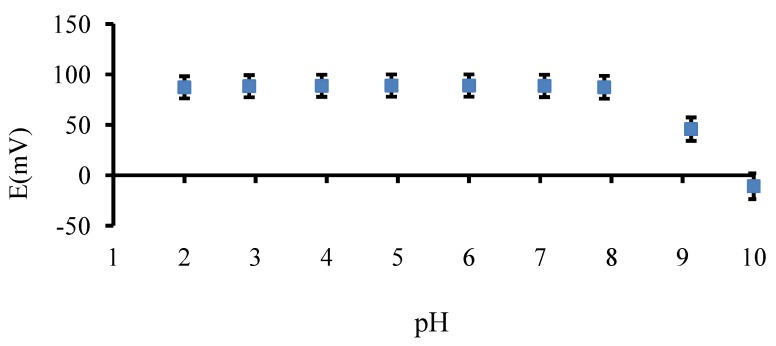
Effect of pH on the potential response of lidocaine sensors. Mean ± SD values are shown (*n* = 3).

To exemplify the pH dependence of electrodes, a constant 1.0 × 10^−3^ M lidocaine hydrochloride solution was measured over the pH range of 2.0–10.0. The pH values were adjusted by adding small volumes of 1.0 M sodium hydroxide or 1.0 M hydrochloric acid to the solution. As Figure 4 shows, the potential response remained almost constant over the pH range of 2.0–8.0. This can be considered as working pH range of the electrodes. The drastic decrease of the potential response at pH > 8.0 was due to the decrease in the protonated form of lidocaine hydrochloride.

#### 3.1.4. Potentiometric Selectivity

To demonstrate the potentiometric selectivity of the Ld^+^ electrodes, the effect of interfering cations were investigated by separate solution method [22]. The results are summarized in Table 1. All the potentiometric selectivity coefficient(*K*^pot^_Lid,M_) values were lower than 1.0 × 10^−3^, indicating that the concentration of each of the interfering cations should be at least 3 orders of magnitude higher than the Ld^+^ in order to affect the potentiometric response of the electrodes. This means that inorganic cations do not interfere the functioning of the electrodes significantly in this research.

**Table 1:** Potentiometric selectivity coefficient of lidocaine electrodes with various interfering cations (*M^n^*^+^).

*M^n^*^+^	*K*^pot^_Lid,M_
K^+^	5.00 × 10^−4^
Na^+^	7.94 × 10^−5^
Ca^2+^	3.98 × 10^−6^
Mg^2+^	3.16 × 10^−6^

### 3.2. Comparison of Potentiometric and UV Spectrophotometry Methods

#### 3.2.1. Polymer Films

**Figure 5:**
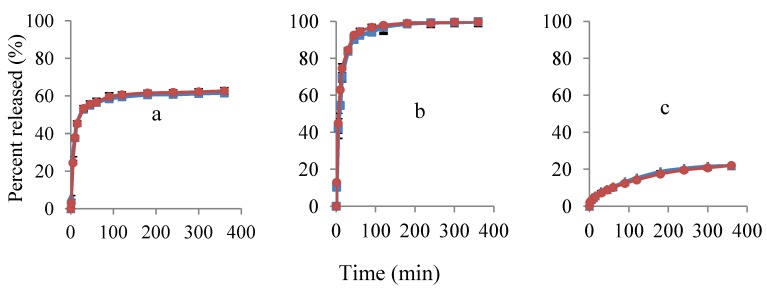
Release profiles of propranolol hydrochloride from solid polymer films with UV (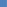) and potentiometric method (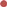). Ratios of EC:HPC in the polymer component are (**a**) 1:1; (**b**) 0:1 and (**c**) 1:0. Mean ± SD values are shown (*n* = 3).

Release profiles of propranolol hydrochloride from the polymer films were studied by the two methods and the results were compared (Figure 5). The data show almost identical release profiles. The release rate of propranolol hydrochloride was considerably higher at the EC:HPC composition of 0:1, which was expected due to the fact that HPC is water soluble. The opposite was observed for the samples containing EC only (Figure 5c). As studied earlier by other researches [23,24,25], EC is known to form a non-swelling, insoluble diffusion matrix to enable sustained release or delayed release profiles for many drug substances. The release from the EC:HPC 1:1 formulation was between the formerly discussed ones and it was similar to the observations made by Kohda *et al*. [26].

#### 3.2.2. Porous Filter Paper Substrates

**Figure 6:**
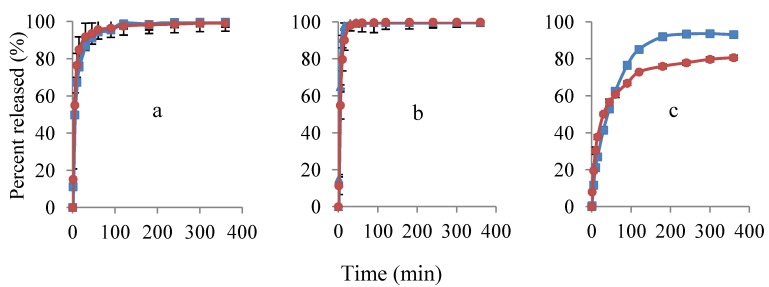
Release profiles of propranolol hydrochloride from porous filter paper substrates with UV (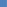) and potentiometric method (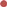). Ratios of EC:HPC in the polymer component are (**a**) 1:1; (**b**) 0:1 and (**c**) 1:0. Mean ± SD values are shown (*n* = 3).

The release profiles of propranolol hydrochloride from filter paper substrates are presented in Figure 6. A similar release profile was detected by both methods. The release of propranolol hydrochloride occurred almost instantly at EC:HPC composition of 0:1. Comparison of the results from Figure 5 and Figure 6 show that the drug release from porous filter paper substrates was substantially higher than its release from the equivalent film formulations. This might be due to the larger drug application area of the filter papers and also the fact that the structure of the filter paper substrate prevented the formation of a complete polymer matrix. This effect can be seen noticeably at EC:HPC composition of 1:1 and 1:0, where the presence of HPC and the nature of the substrate effectively increased the drug permeability and subsequently interfered the formation of the EC rate-controlling matrix.

At EC:HPC composition of 1:0 (Figure 6c), a slight difference in the release profiles (<12%) was observed. This could be related to scattering of the UV light due to the large amount of undissolved cellulose particles and also the interaction of the released Pr^+^ with the components of the cellulose substrate in the solution resulting in less free protonated form of propranolol (lower activity of Pr^+^) was detected by the electrodes.

### 3.3. Study of Lidocaine Hydrochloride Release

**Figure 7:**
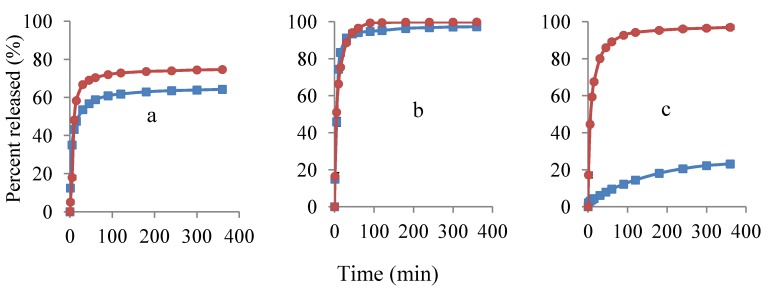
Release profiles of lidocaine hydrochloride from polymer films, using potentiometry with 20% (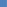) and 40% (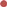) lidocaine in drug-polymer solutions. Ratios of EC:HPC in the polymer component are (**a**) 1:1; (**b**) 0:1 and (**c**) 1:0. Mean ± SD values are shown (*n* = 3).

Figure 7 shows the release profiles of lidocaine hydrochloride from its polymer films. A broad range of drug release patterns could be achieved by changing the drug/polymer blend ratio. Higher and faster release profiles were detected for the samples with 40% API in the composition as opposed to the ones with 20% API. Similar release behavior was previously shown by Kohda *et al*. [26]. The difference in release profiles was considerably high at EC:HPC composition of 0:1, where high drug loading greatly disrupted the complete function of EC matrix to sustain the release of lidocaine. Comparison of the results from Figure 5 and Figure 7 show that formulations containing 20% lidocaine and propranolol had a very similar release behavior for all the blend ratios of polymers.

## 4. Conclusions

The specific design of PVC matrix-type solid-contact electrodes sensitive to propranolol and lidocaine cations was fabricated. The release profiles of APIs were successfully studied by two different methods, namely potentiometry and UV spectrophotometry. The developed ISEs exhibited good performance characteristics to detect the release of both drugs from two different formulations. The life span of the electrodes exceeded six months. Continuous monitoring of the results, very fast response, simple design and the ability to function over a wide pH range, as well as low detection limit, proved the value of the electrodes. The results from both methods were almost identical for different samples. The ISEs provided an alternative and powerful tool to assess the drug release from different drug delivery systems. 
